# Genetically-predicted Pulse Pressure and Risk of Abdominal Aortic Aneurysm: A Mendelian Randomization Analysis

**DOI:** 10.1161/CIRCGEN.121.003575

**Published:** 2022-05-06

**Authors:** Stephen Burgess, Julio A. Chirinos, Scott M. Damrauer, Dipender Gill

**Affiliations:** 1 Medical Research Council Biostatistics Unit, Cambridge Institute of Public Health; 2 Cardiovascular Epidemiology Unit, Dept of Public Health and Primary Care, Univ of Cambridge, Cambridge, United Kingdom; 3 Division of Cardiovascular Medicine, Hospital of the Univ of Pennsylvania, Univ of Pennsylvania Perelman School of Medicine; 4 Depts of Surgery and Genetics, Perelman School of Medicine, Univ of Pennsylvania; 5 Corporal Michael J. Crescenz VA Medical Center, Philadelphia, PA; 6 Dept of Epidemiology & Biostatistics, School of Public Health, Imperial College London; 7 Clinical Pharmacology Group, Pharmacy & Medicines Directorate, St George’s Univ Hospitals NHS Foundation Trust; 8 Clinical Pharmacology & Therapeutics Section, Institute for Infection & Immunity, St George’s, Univ of London, London; 9 Novo Nordisk Research Centre Oxford, Old Road Campus, Oxford, United Kingdom

Pulse pressure (PP), the difference between systolic blood pressure (SBP) and diastolic blood pressure (DBP), arises due to pulsatile ejection of blood from the left ventricle. Previous observational studies have identified an inverse association of PP with aortic diameter^1^, and positive associations of PP with aortic wall stiffness and thickness^2^. However, it is not known whether these associations reflect a causal effect of PP on the risk of AAA, an effect of the aorta on PP, a shared etiology, or confounding from environmental factors.

Here, we investigated the relationship between PP and AAA risk using two-sample Mendelian randomization, which employs genetic variants specifically related to an exposure to define subgroups of the population with different average levels of the exposure. The independent segregation of alleles at conception means these genetically-defined subgroups should not differ systematically with respect to confounding variables, creating a natural experiment analogous to a randomized trial.

Firstly, to investigate the relationship between PP and risk of AAA independent of other blood pressure measures, we performed multivariable Mendelian randomization using the inverse-variance weighted method^3^. In our main analysis, PP and mean arterial pressure (MAP) are the exposures and AAA is the outcome. In secondary analyses, we consider SBP and DBP respectively with PP as exposures in multivariable analyses. AAA events were identified in UK Biobank, a population-based cohort of UK residents aged 40-69 at baseline, based on electronic heath records. Genetic associations with AAA were estimated in 367,586 European ancestry participants (1094 AAA cases) by logistic regression adjusting for age, sex, and 10 principal components of genetic ancestry (to account for potential population stratification). As genetic instruments for blood pressure traits, we selected 258 uncorrelated variants previously associated with blood pressure at a genome-wide level of significance in the International Consortium for Blood Pressure (ICBP)^4^ excluding UK Biobank participants. Genetic associations with blood pressure measures were obtained by meta-analysis of study-specific estimates estimated using linear regression in 299,024 European ancestry participants from the ICBP with UK Biobank participants excluded.

Secondly, to explore the association of genetically-predicted AAA risk with blood pressure measurements, we performed univariable Mendelian randomization analyses with AAA risk as the exposure and blood pressure measures as the outcome. This investigates the relationship between liability to AAA and blood pressure^3^. As instruments for AAA, we considered 24 uncorrelated variants associated with AAA risk at a genome-wide level of significance^5^. For these analyses, genetic associations with blood pressure measures were estimated in UK Biobank using linear regression adjusting for age, sex, and 10 principal components.

All data are publicly available at http://dx.doi.org/10.6084/m9.figshare.17912192. UK Biobank has approval from the North West Multicentre Research Ethics Committee.

Considering the effect of blood pressure on AAA, genetically-predicted MAP was positively associated with AAA risk (estimates are scaled to a 5 mmHg increase in the blood pressure trait; odds ratio [OR] 1.55, 95% confidence interval [CI] 1.21, 2.00; p=0.008), whereas genetically-predicted PP was inversely associated (OR 0.64, 95% CI 0.46, 0.89; p=0.0006). Similar findings were observed in multivariable analyses for SBP and PP, and DBP and PP (Figure). In sex-stratified analyses, inverse associations with PP were similar in magnitude for males and females, although less precise in females with 95% confidence intervals overlapping the null (Figure).

Considering the effect of AAA risk on blood pressure measures, genetically-predicted AAA risk was inversely associated with PP, with a 0.19 mmHg (95% CI 0.08, 0.29; p=0.0006) reduction per unit increase in the log-odds of AAA. An inverse association was also observed with SBP (0.17 mmHg, 95% CI 0.02, 0.32; p=0.024), but not with DBP (-0.01 mmHg, 95% CI -0.10, 0.07; p=0.76) or MAP (0.05 mmHg, 95% CI -0.05, 0.15; p=0.34).

This Mendelian randomization study advances on previous epidemiological investigations to provide evidence supporting a bidirectional inverse relationship between PP and AAA risk, consistent with a shared etiology. Increased stiffness of the aortic wall may underlie this, by raising PP (due to higher SBP and lower DBP) but decreasing risk of AAA (due to less distension of the aortic wall). Another explanation is a threshold effect for case classification, where AAA classification is less likely in individuals with smaller aortas because diagnosis is based on absolute rather than relative diameter. However, estimates were similar in males and females, despite sex differences in aorta size. Limitations of our investigation are that we did not have data on lumen diameter, were not able to assess specific AAA etiologies, and findings may not relate equally to all AAA subtypes. A further limitation is power, particularly for the female-specific analysis.

Our study provides important mechanistic insights regarding the relationship between PP and the risk of AAA. It suggests an inverse relationship between PP and AAA risk, but one that is likely driven by a common underlying mechanism rather than a direct inverse causal effect of PP on AAA risk.

## Figures and Tables

**Figure 1:**
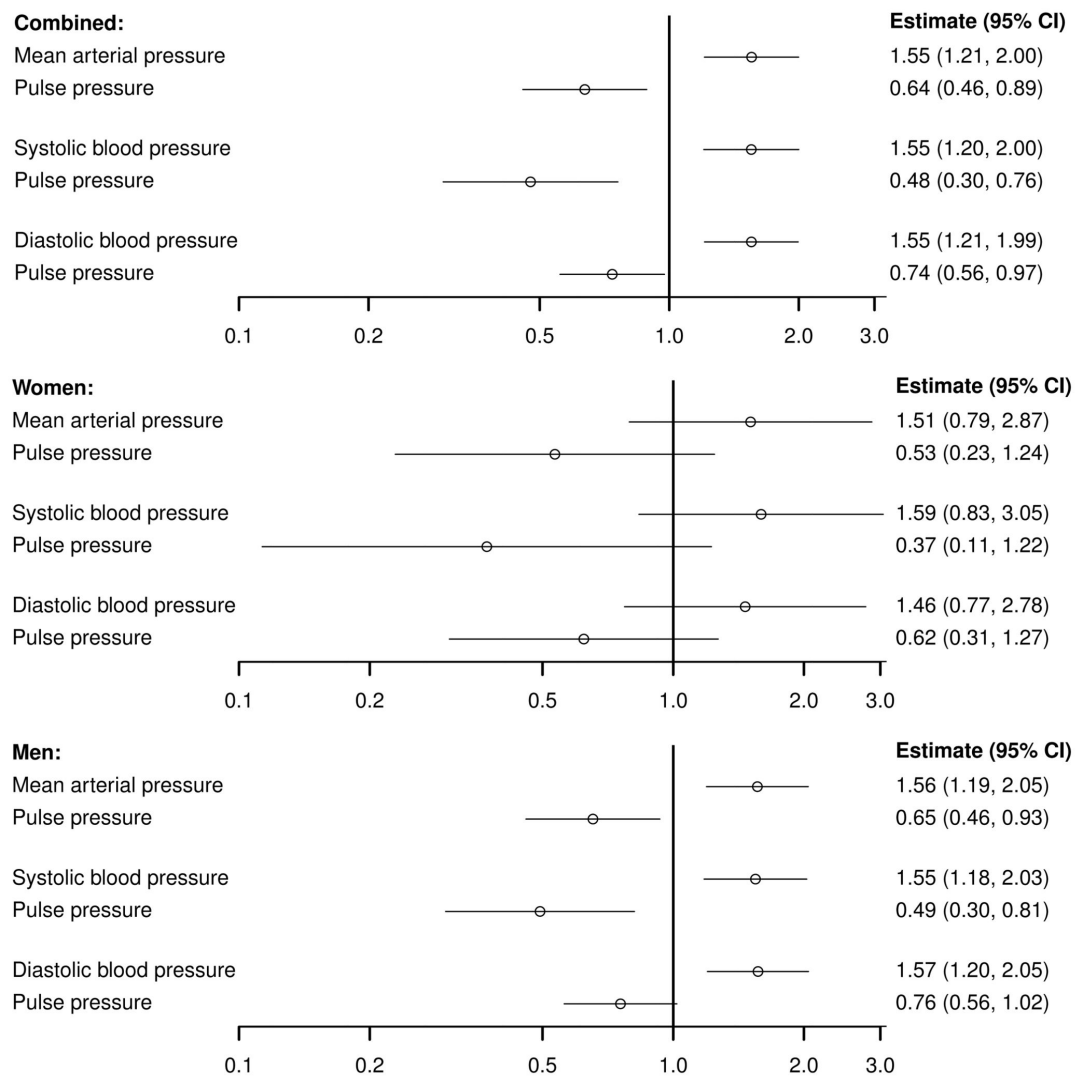
Associations between genetically-predicted blood pressure measures and abdominal aortic aneurysm from 3 separate multivariable Mendelian randomization analyses using combined and sex-stratified genetic associations with abdominal aortic aneurysm risk. Estimates (95% confidence intervals [CI]) represent the odds ratio for disease per 5 mmHg increase in genetically-predicted levels of the blood pressure trait. Separate multivariable analyses were performed for three choices of exposure variables: mean arterial pressure and pulse pressure; systolic blood pressure and pulse pressure; and diastolic blood pressure and pulse pressure. AAA was defined from hospital episode statistics and death certificates using International Classification of Disease coding (9th edition: 441.3 or 441.4, 10th edition I71.3 or I71.4) or hospital procedure coding (Office of Population Censuses and Surveys code: L19.4 or L19.5).

## Nonstandard Abbreviations and Acronyms

AAA: abdominal aortic aneurysm
DBP: diastolic blood pressure
ICBP: International Consortium for Blood Pressure
MAP: mean arterial pressure
PP: pulse pressure
